# The Predictive Factors for Post-Traumatic Hip Osteoarthritis After Surgical Treatment of Acetabular Fractures: A Retrospective Cohort of 130 Patients

**DOI:** 10.3390/jcm15145444

**Published:** 2026-07-11

**Authors:** Onur Mercan, Nevzat Gönder, Mustafa Işık

**Affiliations:** 1 Department of Orthopaedics and Traumatology, T.C. Ministry of Health Birecik State Hospital, Şanlıurfa 63400, Turkey; mercan.md@gmail.com; 2 Department of Orthopaedics and Traumatology, Gaziantep University Faculty of Medicine, Gaziantep 27310, Turkey

**Keywords:** acetabular fracture, post-traumatic osteoarthritis, Judet–Letournel classification, matta criteria, Kocher–Langenbeck approach

## Abstract

**Background/Objectives:** Post-traumatic hip osteoarthritis (PTHOA) remains the principal long-term complication after operatively treated acetabular fractures. The relative weight of patient-, injury-, and surgeon-dependent variables that drive its development is still debated. We aimed to identify independent predictors of PTHOA after open reduction and internal fixation (ORIF) of acetabular fractures. **Methods:** We retrospectively reviewed 130 consecutive patients who underwent ORIF for an acetabular fracture at a single tertiary trauma centre between November 2012 and January 2023 and had a minimum follow-up of 24 months. Fractures were classified using the Judet–Letournel system; reduction quality was graded according to Matta. PTHOA was assessed at the latest follow-up using both the Tönnis and Kellgren–Lawrence (KL) systems. Patients were stratified into three groups: no OA, mild OA (Tönnis 1/KL 1–2), and advanced OA (Tönnis 2–3/KL 3–4). Univariable and multivariable analyses identified factors independently associated with advanced OA. **Results:** The cohort included 106 men (81.5%) and 24 women (18.5%) with a mean age of 40.7 ± 16.3 years and a mean follow-up of 3.4 years. Advanced PTHOA developed in 43 patients (33.1%); 13 (10.0%) underwent THA. On multivariable analysis, prolonged time-to-surgery (>6 days; *p* = 0.001), associated posterior-wall–transverse fracture (*p* = 0.038), use of the Kocher–Langenbeck (K-L) approach (*p* = 0.038), and non-anatomic Matta reduction (*p* < 0.001) were independently associated with advanced PTHOA. Age ≥ 40 years was significant on univariable analysis (*p* = 0.002) but not after adjustment. **Conclusions:** Reduction quality, associated posterior-wall–transverse fracture morphology, surgical timing, and choice of approach are the principal determinants of PTHOA after acetabular ORIF. Early surgery and meticulous anatomic reduction should remain the cornerstones of management.

## 1. Introduction

Acetabular fractures are intra-articular injuries that disrupt the load-bearing surface of the hip and, by virtue of the complex three-dimensional anatomy of the acetabulum, remain one of the most challenging problems in orthopaedic trauma surgery [1,2]. In young adults, they typically result from high-energy mechanisms such as motor vehicle collisions and falls from height, whereas in elderly patients, they may follow comparatively low-energy trauma due to compromised bone quality [3].

Open reduction and internal fixation (ORIF) is the standard of care for displaced fractures and unstable patterns, with the goals of restoring articular congruity, achieving stable fixation, and preventing post-traumatic hip osteoarthritis (PTHOA) [4]. Despite optimal surgical management, PTHOA remains the most frequent and most disabling long-term complication, occurring in approximately 20–30% of patients at mid-term follow-up and being the principal indication for conversion to total hip arthroplasty (THA) [5,6,7].

A range of variables has been implicated in PTHOA development, including patient age, time elapsed from injury to surgery, fracture pattern, associated femoral head injury or hip dislocation, and, most consistently, the quality of articular reduction [8,9,10]. However, the relative contribution of each factor varies between series, and the interactions between fracture pattern and choice of surgical approach are particularly difficult to disentangle. Understanding these predictors is clinically important: it enables risk stratification, informs preoperative counselling, and identifies modifiable targets to improve long-term outcomes.

The aim of this study was to identify independent predictors of PTHOA in patients undergoing ORIF for acetabular fractures, with particular attention to modifiable variables such as surgical timing, choice of approach, and reduction quality.

## 2. Materials and Methods

### 2.1. Study Design

We performed a single-centre, retrospective cohort study of consecutive patients who underwent ORIF for an acetabular fracture at the Department of Orthopaedics and Traumatology, Gaziantep University Faculty of Medicine, between November 2012 and January 2023. The study was approved by the Gaziantep University Clinical Research Ethics Committee (approval no. 2025/222, dated 6 August 2025) and was conducted in accordance with the Declaration of Helsinki. Informed consent was obtained from all subjects involved in the study. The study is reported in accordance with the STROBE statement.

### 2.2. Patient Selection

Inclusion criteria were (i) acetabular fracture treated by ORIF; (ii) minimum clinical and radiographic follow-up of 24 months; (iii) availability of pre-operative, immediate post-operative, and latest follow-up imaging; and (iv) absence of pre-existing osteoarthritis or prior hip surgery on the affected side. Patients managed non-operatively, those with follow-up shorter than 24 months, those with deep post-operative wound infection, and those who underwent additional ipsilateral hip procedures unrelated to PTHOA during the follow-up period were excluded.

### 2.3. Pre-Operative Assessment and Radiographic Classification

All patients underwent standardised initial assessment in the emergency department including anteroposterior (AP) pelvis radiographs and Judet (iliac and obturator oblique) views, supplemented by computed tomography (CT) once haemodynamically stable. Concomitant hip dislocation was reduced under sedation in the emergency department or under general anaesthesia in the operating room as soon as possible after presentation.

Fractures were classified according to the Judet–Letournel system using a standardised, algorithm-based approach to minimise observer subjectivity, drawing on the iliopectineal and ilioischial lines, anterior and posterior walls, the integrity of the obturator foramen, the presence of an ischial ramus or iliac wing extension, and pathognomonic radiographic signs [11,12,13].

### 2.4. Surgical Procedure

Surgical approach was selected based on fracture pattern, associated injuries, and surgeon preference, in accordance with established Judet–Letournel principles [11]. Available approaches were Kocher–Langenbeck (K-L), modified Stoppa, ilioinguinal, and combined anterior–posterior exposures. All procedures were performed or supervised by senior pelvic trauma surgeons. Anatomic reduction was the goal in every case, with stable internal fixation using contoured pelvic reconstruction plates and screws. All patients received cefazolin 1 g for at least 24 h peri-operatively and pharmacological venous thromboembolism prophylaxis with low-molecular-weight heparin unless contraindicated.

Post-operatively, in-bed exercises were initiated as early as tolerated, followed by non-weight-bearing mobilisation. Partial weight bearing was permitted from week six and full weight bearing typically between weeks 10 and 12, depending on radiographic union.

### 2.5. Outcome Measures

The primary outcome was the development of advanced PTHOA at the latest follow-up. Reduction quality on the immediate post-operative radiograph was graded according to Matta criteria (anatomic, <1 mm displacement; satisfactory, 1–3 mm; poor, >3 mm) [4]. At follow-up, hip osteoarthritis was graded independently using both the Tönnis and KL classifications [14,15]. All follow-up radiographs were graded independently by two orthopaedic surgeons who were blinded to the intra-operative findings and to the reduction grade; disagreements were resolved by consensus, and where consensus could not be reached the higher grade was assigned. Patients were then categorised as (i) no OA (Tönnis 0/KL 0); (ii) mild OA (Tönnis 1/KL 1–2); or (iii) advanced OA (Tönnis 2–3/KL 3–4). Patients who underwent THA for symptomatic PTHOA during follow-up were assigned to the advanced OA group regardless of the most recent radiographic grade.

The following variables were extracted: age, sex, mechanism of injury, associated skeletal injuries, time from injury to surgery, Judet–Letournel fracture type, presence of hip dislocation at presentation, surgical approach, Matta reduction quality, follow-up duration, and conversion to THA.

### 2.6. Statistical Analysis

Analyses were performed using IBM SPSS Statistics version 23 (IBM Corp., Armonk, NY, USA). Continuous variables were tested for normality (Kolmogorov–Smirnov/Shapiro–Wilk and skewness–kurtosis) and reported as mean ± standard deviation or median (range), as appropriate. Categorical variables are expressed as counts and percentages. The correlation between Tönnis and KL grades was assessed with Pearson’s correlation; given a correlation coefficient of 0.981, the Tönnis-based three-tier classification was used as the primary outcome variable. Group comparisons used the chi-square or Fisher’s exact test for categorical variables, one-way ANOVA for normally distributed continuous variables, and the Kruskal–Wallis test otherwise. Variables with *p* < 0.05 on univariable analysis were entered into a multivariable logistic regression model (advanced OA vs. no/mild OA) to identify independent predictors. For each variable, the strength of association was expressed as an odds ratio (OR) with a 95% confidence interval (CI); unadjusted ORs were obtained from univariable analysis and adjusted ORs (aOR) from the multivariable logistic regression model. A two-sided *p* value of <0.05 was considered statistically significant.

## 3. Results

One hundred and thirty patients (106 men, 81.5%; 24 women, 18.5%) with a mean age of 40.7 ± 16.3 years (range, 13–80) were analysed. Mean follow-up was 3.4 years (range, 2–12). The most frequent mechanisms were motor vehicle accidents (57.7%; 28.5% in-vehicle, 15.4% pedestrian, 13.7% motorcycle), followed by falls from height (30.8%) and crush injuries (11.5%). Eighty patients (61.5%) had at least one additional skeletal injury, most commonly involving the pelvic ring (26.2%), sacrum (14.3%), spine (11.1%), femur (10.3%), and patella (10.3%). Ipsilateral hip dislocation was present at admission in 19 patients (14.6%). Patient demographics are summarised in Table 1.

According to the Judet–Letournel classification, 86 patients (66.2%) had elementary fractures and 44 (33.8%) had associated patterns. The most common types were posterior wall (n = 28, 21.5%), both-column (n = 20, 15.4%), transverse (n = 18, 13.8%), anterior wall (n = 16, 12.3%), associated posterior wall–transverse (n = 14, 10.8%), anterior column (n = 14, 10.8%), posterior column (n = 10, 7.7%), T-type (n = 9, 6.9%), and anterior column–posterior hemitransverse (n = 4, 3.1%) fractures.

Mean time from injury to surgery was 5.9 ± 4.7 days (range, 1–30); 90 patients (69.2%) underwent surgery within six days. Surgical approaches included isolated K-L (n = 39, 30.0%), isolated modified Stoppa (n = 39, 30.0%), combined K-L and modified Stoppa (n = 35, 26.9%), isolated ilioinguinal (n = 13, 10.0%), and combined K-L–ilioinguinal (n = 4, 3.1%). According to Matta criteria, reduction was anatomic in 77 patients (59.2%), satisfactory in 39 (30.0%), and poor in 14 (10.8%).

At the latest follow-up, 69 patients (53.1%) had no radiographic OA, 18 (13.8%) had mild OA, and 43 (33.1%) had advanced OA. Thirteen patients (10.0%) underwent conversion to THA. Tönnis and KL grades were highly correlated (Pearson r = 0.981).

### 3.1. Univariable Predictors of Advanced PTHOA

Patient age was significantly associated with PTHOA: advanced OA developed in 31 of 65 patients (47.6%) aged ≥ 40 years versus 12 of 65 (18.4%) aged < 40 years (*p* = 0.002) (OR 4.03, 95% CI 1.82–8.90). Sex (*p* = 0.18) and mechanism of injury (*p* = 0.43) were not associated with PTHOA.

Hip dislocation at presentation was associated with a higher rate of advanced PTHOA (11/19, 57.9%) compared with patients without dislocation (32/111, 28.8%; *p* = 0.04) (OR 3.40, 95% CI 1.25–9.22).

Time to surgery differed significantly between groups (mean 7.9 ± 5.8 days in the advanced OA group vs. 5.0 ± 3.9 days in the no-OA group; *p* < 0.001). Dichotomising at six days, advanced OA occurred in 24 of 40 patients (60.0%) operated after day 6 versus 19 of 90 (21.1%) operated within six days (*p* < 0.001) (OR 5.61, 95% CI 2.49–12.61).

Because the Judet–Letournel categories in Table 2 are mutually exclusive, the posterior wall row represents isolated posterior wall fractures rather than any posterior wall involvement. Isolated posterior wall fractures had a lower crude rate of advanced PTHOA than the cohort average (6/28, 21.4% vs. 43/130, 33.1%; crude OR 0.48, 95% CI 0.18–1.29; *p* = 0.176) and therefore were not considered a risk-increasing predictor. The highest crude odds were observed in posterior column and associated posterior wall–transverse patterns; after multivariable adjustment, only the associated posterior wall–transverse pattern remained independently associated with advanced PTHOA (Table 2).

Surgical approach was significantly associated with PTHOA (*p* < 0.001). Advanced OA developed in 18 of 39 patients (46.2%) treated with isolated K-L, 15 of 35 (42.9%) with combined K-L–Stoppa, 3 of 4 (75.0%) with combined K-L–ilioinguinal, 6 of 39 (15.4%) with isolated modified Stoppa, and 1 of 13 (7.7%) with isolated ilioinguinal. When K-L use (whether isolated or as part of a combined approach, n = 78) was compared with non-K-L approaches (n = 52), advanced OA developed in 36/78 (46.2%) vs. 7/52 (13.5%) patients (*p* < 0.001) (OR 5.51, 95% CI 2.21–13.72). Notably, 11 of the 13 patients (84.6%) who progressed to THA had been treated through a K-L approach.

Matta reduction quality showed the strongest gradient: advanced OA was observed in 13/77 anatomic (16.9%), 17/39 satisfactory (43.6%), and 13/14 poor reductions (92.9%; *p* < 0.001; non-anatomic vs. anatomic reduction: OR 6.42, 95% CI 2.87–14.38) Table 3. 

### 3.2. Multivariable Analysis

On multivariable logistic regression, the variables that remained independently associated with advanced PTHOA were prolonged time to surgery (>6 days), associated posterior wall–transverse fracture, use of the K-L approach, and non-anatomic Matta reduction. Patient age ≥ 40 years and presence of hip dislocation lost statistical significance after adjustment (Table 4).

## 4. Discussion

In this single-centre cohort of 130 patients followed for a minimum of two years after ORIF of an acetabular fracture, advanced PTHOA developed in approximately one-third of patients, and 10% required conversion to THA. The principal independent predictors of advanced PTHOA were non-anatomic reduction, associated posterior wall–transverse fracture morphology, the use of the Kocher–Langenbeck approach, and prolonged time to surgery.

The overall incidence of PTHOA in our series (33%) is consistent with rates of 18–30% reported in contemporary cohorts [5,7,16]. Tannast et al. found a 21% rate at a mean follow-up of more than seven years in 810 patients, while Clarke-Jenssen et al. and the seminal Letournel series both reported approximately 18% at long-term follow-up [5,6,17]. Slightly higher rates in older cohorts (~28%) match our observation that patients aged ≥40 years were nearly three times more likely to develop advanced OA on univariable analysis [18,19].

Although age was significant in univariable analysis, it was not retained as an independent predictor after adjustment, likely because it co-varies with bone quality, comminution, and reduction quality. This finding is congruent with the wider literature: while several authors consider advanced age an independent risk factor, others have shown that, when articular reduction is anatomic, age has only a limited effect on outcome [4,20,21]. Our data therefore support the view that age is best regarded as a surrogate for fracture complexity and biological factors that act jointly to compromise the joint.

Surgical timing emerged as a strong, modifiable predictor in our cohort: every additional day of delay was associated with a higher risk of advanced PTHOA. Among patients operated within six days, only 21% developed advanced OA, compared with 60% of those operated later. This is consistent with Letournel’s original observation that 80.7% of patients operated within three weeks achieved excellent reduction and with more recent reports linking delays beyond two weeks to a near five-fold increase in poor outcomes [17,22,23]. Beyond purely technical considerations (callus formation, soft-tissue contracture, and fragment resorption), prolonged delay also exposes the articular cartilage to repeated mechanical trauma against incongruent surfaces. Once the patient is haemodynamically stable, definitive fixation should not be unduly postponed.

The role of fracture pattern remains nuanced. Although the global *p* value for fracture type was clinically informative, the elementary-versus-associated dichotomy was not, and only the associated posterior wall–transverse fracture pattern independently predicted advanced PTHOA after adjustment. Posterior wall involvement is the most consistently reported risk factor across the literature; Matta and Mehne attributed this to the diffuse cartilage damage produced by impaction of the femoral head against the wall fragment, and Kreder et al. showed that even anatomic reduction did not abolish the risk of OA in comminuted posterior wall patterns [24,25]. Long-term survivorship analyses confirm a 5–20-year native hip survival of 76–85% in posterior wall fractures [5,26,27]. Transverse fractures, by traversing the weight-bearing dome, also carry a particular risk for cartilage failure, especially when associated with a posterior wall component [28].

Our finding that the Kocher–Langenbeck approach was independently associated with advanced PTHOA must be interpreted with caution. K-L is preferentially used for posterior wall, posterior column, and selected transverse and T-type fractures, patterns with intrinsically poor prognosis, and the association may therefore reflect confounding by indication. Even so, the magnitude of the effect (46% advanced OA with K-L involvement vs. 14% with anterior approaches), and the observation that 85% of patients requiring THA had been treated through a K-L approach, may nonetheless indicate a contribution of the approach itself: posterior dissection through the short external rotators and capsule may compromise femoral head perfusion and gluteal mechanics, and contribute to articular dysfunction independently of the fracture pattern [29,30]. Nevertheless, because the choice of the Kocher–Langenbeck approach is largely dictated by posterior wall and column involvement, its apparent effect cannot be cleanly separated from the severity of the underlying fracture pattern in a non-randomised cohort. A substantial part of the observed association with the K-L approach therefore probably reflects the fracture morphology that mandates a posterior exposure rather than the exposure per se, and this finding should be regarded as hypothesis-generating rather than as evidence of a causal, approach-specific effect. A direct, pattern-matched comparison with alternative posterior or combined approaches in higher-volume centres would be a valuable next step.

Reduction quality showed the strongest and most consistent gradient in our series, with advanced OA rates of 17%, 44%, and 93% in anatomic, satisfactory, and poor reductions, respectively. This directly mirrors the seminal data of Matta (88% good/excellent results after anatomic reduction, falling to 48% with poor reduction) and Kreder et al. (82% vs. 48% good outcomes for ≤2 mm vs. >2 mm step-off) [4,25]. The implication is unambiguous: anatomic reduction is the single most important variable under the surgeon’s control.

Hip dislocation at presentation was associated with PTHOA on univariable analysis but did not survive multivariable adjustment, likely because it strongly co-varies with posterior wall pattern and femoral head injury. Nevertheless, the well-established association between dislocation and chondral damage supports the principle that emergent closed reduction should remain the standard of care to limit cartilage injury, irrespective of definitive surgical timing [5,28].

This study has several limitations. First, it is a retrospective, single-centre analysis subject to the inherent biases of such designs, including selection bias and the possibility of unmeasured confounding. Second, although follow-up exceeded two years in all patients, the mean follow-up of 3.4 years may underestimate the true long-term incidence of PTHOA, which can develop 15–20 years after injury [11]. Because post-traumatic osteoarthritis may continue to progress for up to two decades after the index injury, the predictors identified here relate primarily to early- and mid-term PTHOA; both the cumulative incidence and the magnitude of the reported associations may therefore change with longer surveillance, and our findings should be interpreted with this limitation in mind. Third, functional outcome measures (e.g., Harris Hip Score, Merle d’Aubigné–Postel) were not consistently available and could not be analysed. Fourth, surgical approach was not randomised; the association with K-L should therefore be interpreted as hypothesis-generating. Fifth, the relatively small number of patients in some Judet–Letournel subgroups limits the statistical power for less common patterns. In addition, although radiographic grading was performed independently by two observers, formal interobserver reliability (e.g., weighted kappa) for the Tönnis and Kellgren–Lawrence classifications was not quantified, which may introduce a degree of measurement variability. Finally, post-operative CT was not routinely performed, and reduction quality was therefore assessed on plain radiographs, which is known to under-estimate residual articular step-off compared with CT.

## 5. Conclusions

Advanced PTHOA developed in approximately one-third of patients within a mean of 3.4 years after ORIF of an acetabular fracture in this cohort, and 10% required conversion to total hip arthroplasty. Predictors of advanced PTHOA were prolonged time to surgery, associated posterior wall–transverse fracture morphology, the use of the Kocher–Langenbeck approach, and non-anatomic reduction (although the association with the Kocher–Langenbeck approach should be interpreted cautiously, as it is closely linked to the posterior fracture patterns for which this exposure is indicated). While fracture pattern is non-modifiable, surgical timing, choice of approach, and, most importantly, the quality of articular reduction are under the surgeon’s control. Prompt definitive fixation once the patient is stable, careful pattern-specific selection of the surgical exposure, and uncompromising attention to anatomic reduction should remain the cornerstones of management. Patients with high-risk fracture patterns and/or poor reduction warrant closer surveillance and earlier counselling regarding the possibility of conversion to arthroplasty.

## Figures and Tables

**Table 1:** Demographic and injury characteristics of the 130 patients.

Variable	Value
Number of patients	130
Sex, n (%)	
Male	106 (81.5%)
Female	24 (18.5%)
Age, mean ± SD (years)	40.7 ± 16.3 (range 13–80)
Age ≥ 40 years, n (%)	65 (50.0%)
Mechanism of injury, n (%)	
Motor vehicle accident	37 (28.5%)
Pedestrian struck	20 (15.4%)
Motorcycle accident	17 (13.1%)
Fall from height	40 (30.8%)
Crush injury	15 (11.5%)
Hip dislocation at presentation, n (%)	19 (14.6%)
Associated skeletal injury, n (%)	80 (61.5%)
Time from injury to surgery, mean ± SD (days)	5.9 ± 4.7 (range 1–30)
Operated within 6 days, n (%)	90 (69.2%)
Mean follow-up, years (range)	3.4 (2–12)

**Table 2:** Distribution and crude odds of advanced post-traumatic hip osteoarthritis according to mutually exclusive Judet–Letournel fracture type.

Fracture Type	n	Advanced OA, n (%)	*p*-Value	Crude OR (95% CI)
Posterior wall	28	6 (21.4%)	0.176	0.48 (0.18–1.29)
Posterior column	10	6 (60.0%)	0.081	3.36 (0.90–12.64)
Anterior wall	16	3 (18.8%)	0.261	0.43 (0.11–1.59)
Anterior column	14	1 (7.1%)	0.034	0.14 (0.02–1.07)
Transverse	18	8 (44.4%)	0.289	1.76 (0.64–4.84)
Posterior wall + transverse	14	8 (57.1%)	0.068	3.09 (1.00–9.56)
Anterior column + posterior hemitransverse	4	0 (0.0%)	0.301	0.21 (0.01–4.05)
T-type	9	2 (22.2%)	0.717	0.56 (0.11–2.81)
Both-column	20	9 (45.0%)	0.301	1.83 (0.69–4.82)

**Table 3:** Distribution of osteoarthritis grade at latest follow-up according to Matta reduction quality.

Matta Reduction	n (%)	No OA, n (%)	Mild OA, n (%)	Advanced OA, n (%)
Anatomic (<1 mm)	77 (59.2%)	57 (74.0%)	7 (9.1%)	13 (16.9%)
Satisfactory (1–3 mm)	39 (30.0%)	12 (30.8%)	10 (25.6%)	17 (43.6%)
Poor (>3 mm)	14 (10.8%)	0 (0.0%)	1 (7.1%)	13 (92.9%)
Total	130 (100%)	69 (53.1%)	18 (13.8%)	43 (33.1%)

**Table 4:** Univariable and multivariable analysis of predictors of advanced post-traumatic hip osteoarthritis.

Variable	Univariable OR (95% CI)	Univariable *p*	Adjusted OR (95% CI)	Adjusted *p*	Independent Predictor
Age ≥ 40 years	4.03 (1.82–8.90)	0.002	1.82 (0.71–4.67)	0.21	No
Male sex	—	0.18	—	—	No
Mechanism of injury	—	0.43	—	—	No
Hip dislocation at presentation	3.40 (1.25–9.22)	0.04	1.58 (0.48–5.22)	0.45	No
Time to surgery > 6 days	5.61 (2.49–12.61)	<0.001	4.68 (1.82–12.05)	0.001	Yes
Posterior wall fracture	0.48 (0.18–1.29)	0.176	0.71 (0.22–2.31)	0.57	No
Transverse fracture	1.76 (0.64–4.84)	0.289	1.54 (0.49–4.87)	0.46	No
Posterior wall + transverse	3.09 (1.00–9.56)	0.068	3.96 (1.08–14.54)	0.038	Yes
Kocher–Langenbeck approach (any use)	5.51 (2.21–13.72)	<0.001	3.21 (1.07–9.66)	0.038	Yes
Non-anatomic Matta reduction	6.42 (2.87–14.38)	<0.001	6.12 (2.32–16.15)	<0.001	Yes

OR, odds ratio; CI, confidence interval. The fracture-pattern rows in Table 2 and Table 4 are coded as mutually exclusive Judet–Letournel categories. Therefore, the posterior wall row denotes isolated posterior wall fracture and not “any posterior wall involvement”. Adjusted estimates are reported from the multivariable logistic regression model for advanced OA versus no/mild OA.

## Data Availability

Data available on request due to restrictions (e.g., privacy, legal or ethical reasons).

## Abbreviations

The following abbreviations are used in this manuscript:

ANOVA	Analysis of variance
AP	Anteroposterior
CT	Computed tomography
KL	Kellgren–Lawrence (classification)
K-L	Kocher–Langenbeck (approach)
OA	Osteoarthritis
ORIF	Open reduction and internal fixation
PTHOA	Post-traumatic hip osteoarthritis
SD	Standard deviation
SPSS	Statistical Package for the Social Sciences
STROBE	Strengthening the Reporting of Observational Studies in Epidemiology
THA	Total hip arthroplasty
